# Genome-wide linkage analyses identify *Hfhl1* and *Hfhl3* with frequency-specific effects on the hearing spectrum of NIH Swiss mice

**DOI:** 10.1186/1471-2156-13-32

**Published:** 2012-04-27

**Authors:** James M Keller, Konrad Noben-Trauth

**Affiliations:** 1 Section on Neurogenetics, Laboratory of Molecular Biology, National Institute on Deafness and Other Communication Disorders, National Institutes of Health, 5 Research Court, Rockville, MD, 20850, USA

**Keywords:** NIH Swiss, Sensorineural hearing loss, Quantitative trait loci analyses, Tonotopy, DPOAE

## Abstract

**Background:**

The mammalian cochlea receives and analyzes sound at specific places along the cochlea coil, commonly referred to as the tonotopic map. Although much is known about the cell-level molecular defects responsible for severe hearing loss, the genetics responsible for less severe and frequency-specific hearing loss remains unclear. We recently identified quantitative trait loci (QTLs) *Hfhl1* and *Hfhl2* that affect high-frequency hearing loss in NIH Swiss mice. Here we used 2f1-f2 distortion product otoacoustic emissions (DPOAE) measurements to refine the hearing loss phenotype. We crossed the high frequency hearing loss (HFHL) line of NIH Swiss mice to three different inbred strains and performed linkage analysis on the DPOAE data obtained from the second-generation populations.

**Results:**

We identified a QTL of moderate effect on chromosome 7 that affected 2f1-f2 emissions intensities (*Hfhl1*), confirming the results of our previous study that used auditory brainstem response (ABR) thresholds to identify QTLs affecting HFHL. We also identified a novel significant QTL on chromosome 9 (*Hfhl3*) with moderate effects on 2f1-f2 emissions intensities. By partitioning the DPOAE data into frequency subsets, we determined that *Hfhl1* and *Hfhl3* affect hearing primarily at frequencies above 24 kHz and 35 kHz, respectively. Furthermore, we uncovered additional QTLs with small effects on isolated portions of the DPOAE spectrum.

**Conclusions:**

This study identifies QTLs with effects that are isolated to limited portions of the frequency map. Our results support the hypothesis that frequency-specific hearing loss results from variation in gene activity along the cochlear partition and suggest a strategy for creating a map of cochlear genes that influence differences in hearing sensitivity and/or vulnerability in restricted portions of the cochlea.

## Background

Mammals discriminate sound energy over a wide range of intensities and frequencies due to adaptations that have produced a cochlea that amplifies and attenuates input in a non-linear and frequency-specific manner. The astounding versatility of the mammalian cochlea is possible because its physiological and biomechanical characteristics change from the base to the apex to produce a gradient of frequency-specific regions referred to as the tonotopic map [1]. The mammalian outer hair cells (OHCs), which are capable of changing length in response to mechanical forces that deflect their stereocilia and alter their membrane potential, play a primary role in tonotopy [2,3]. As might be expected, changes in a number of OHC characteristics parallel changes in frequency sensitivity. For example, OHCs are longer and have longer stereocilia at the apex of the cochlea than at the base [1]. Additionally, the expression of genes coding for ion channels and other molecules thought to be important in OHC function exhibit tonotopic gradients [4]. Graded changes in ion channel structure or composition provides the most parsimonious explanation for the discovery that the conductance of the cochlear mechanoelectrical transduction (MET) channels changes tonotopically, at least in turtles [5]. Perturbations in the expression or function of a number of the genes necessary for OHC activity have been shown to impair hearing [6], but the mechanisms responsible for coordinating and differentiating expression of these genes along the mammalian organ of Corti are yet to be determined.

Many loci have been linked to hearing deficits in humans (http://hereditaryhearingloss.org/) and mice (http://hearingimpairment.jax.org/). However, many other loci probably remain undiscovered, including those that contribute to tonotopy. Although evidence suggests that the primary morphogen Sonic hedgehog (Shh) plays a key role in initiating tonotopic development by establishing the gradient of Gli transcription factors that first defines the basal and apical identities of the cochlear duct [7], the downstream molecules responsible for establishing and maintaining the proper physiological characteristics required for tonotopy still need to be determined. Identifying the genes responsible for this phenomenon may be necessary to understand less severe, but more common, forms of human hearing impairment, including presbycusis and other forms of hearing loss that preferentially affect particular frequency ranges. Despite recent advances, human genome wide association studies (GWAS) remain much less powerful for detecting genes involved in complex, multifactorial diseases than for diseases with simple Mendelian expression, indicating that mouse models may be critical for identifying genes related to tonotopy.

NIH Swiss mice are genetically heterogeneous [8] and have significantly variable 32 kHz ABR thresholds [9]. Recently, we sought to determine the genetic basis for hearing variation in NIH Swiss outbred mice via selective breeding and linkage mapping [10]. We were able to locate at least two QTLs (Quantitative Trait Locus), *Hfhl1* on chromosome 7 and *Hfhl2* on chromosome 8, that affect 32 kHz ABR thresholds but not lower frequency thresholds [10]. The results of that study suggested that the line of mice selected for high-frequency hearing impairment would be useful for studying genes that differentially affect the base and the apex of the cochlea. Here, we performed linkage mapping using additional NIH Swiss crosses to verify the previously discovered QTLs. We also made use of the quantitative data obtained from DPOAE measurements to expand our analysis of the genetics of the novel high-frequency hearing loss phenotype. Finally, since functionally and developmentally related components of a biological feature are expected to be more integrated than less related components [11], we expected topologically proximal regions of the organ of Corti to be more integrated than more distally spaced regions. So, we examined the relationship between DPOAE intensities produced in response to a range of input frequencies across the murine auditory spectrum (i.e. produced at different tonotopic locations). We hypothesized that the developmental and structural relationships between different cochlear regions would translate to functional relationships that would be detectable as correlations in the DPOAE data that vary based on relative tonotopic position and are influenced by genetic differences within the test populations.

## Results

**Figure 1:**
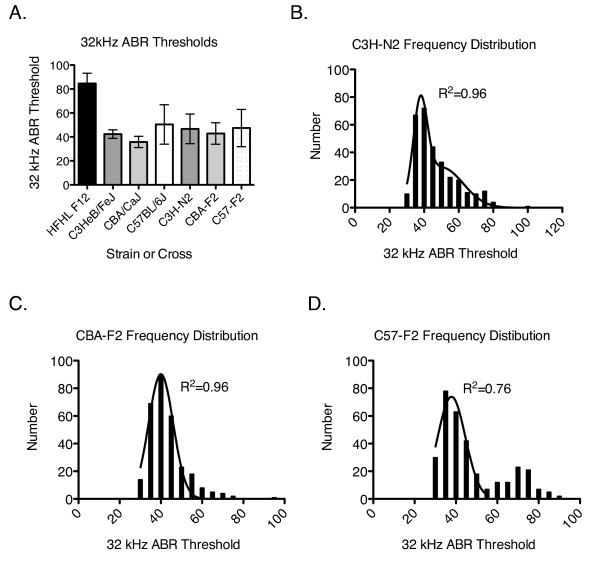
**Auditory brainstem responses.** (**A**) ABR thresholds in dB SPL for 32 kHz pure tone stimuli in the parental and second-generation (N2 and F2) populations. (**B-D**) Threshold distributions for (**B**) C3H-N2, (**C**) CBA-F2, and (**D**) C57-F2 populations. (**B**) The C3H-N2 frequency distribution was produced using data presented previously [10] and is reproduced here for clarification and presentation purposes. This distribution is best fit by the sum of two Gaussian distributions (Goodness-of-fit R^2^ = 0.96, n = 306). (**C**) The CBA-F2 frequency distribution was fitted using a Gaussian distribution (Goodness-of fit R^2^ = 0.96, n = 294). (**D**) The C57-F2 frequency distribution appears to be bi-modal but only the first peak of the curve could be fitted initially (Goodness-of-fit R^2^ = 0.76, n = 320). When the data from individuals with 32 kHz ABR thresholds 60 dB SPL or greater was analyzed separately, the second peak fit a Gaussian distribution (Goodness-of-fit R^2^ = 0.85, n = 82).

To obtain a preliminary assessment of high-frequency hearing, we recorded 32 kHz ABR thresholds for HFHL, C3HeB/FeJ, CBA/CaJ, C57BL/6J, C3H-N2, CBA-F2, and C57-F2 mice at 4–8 weeks of age (Figure 1A, Table 1). As expected, the HFHL mice had 32 kHz ABR thresholds significantly higher than all other groups (*p* < 0.0001). However, although the C3H-N2 and CBA-F2 populations appear to have higher hearing thresholds than the respective normal-hearing parental lines, these differences were not statistically significant. Similarly, the C57-F2 population had ABR thresholds that were not different from the parental C57BL/6J strain. On the other hand, variation in hearing thresholds was greater for the C3H-N2 and CBA-F2 populations than for their respective normal hearing parental strains (*p* < 0.0001) but not the HFHL line.

**Table 1:** High Frequency Phenotypes by Line or Cross

			**32 kHz ABR**		**HFEA**	
**Line/Cross**	**Age (wks)**	**n**	**mean**	**SD**	**mean**	**SD**
HFHL-F12	8	12	84.6	8.6	2.6	2.0
C3HeB/FeJ	8–10	17	42.4	3.6	17.9	3.6
CBA/CaJ	8	12	35.8	4.7	18.6	4.0
C57BL/6J	8	12	50.4	16.4	13.4	6.9
NIHxC3H F1	8	21	37.4	7.0	19.3	2.4
NIHxC3H N2	8	306	46.7	12.4	11.2	6.4
NIHxCBA F2	8	294	42.9	9.0	16.8	6.5
NIHxC57 F2	8	320	47.4	15.5	14.9	8.6

wks, weeks; n, number of animals tested; SD, standard deviation; HFEA, High Frequency Emissions Average.

To investigate the genetic basis underlying differences in hearing in the C3H-N2 (n = 305), CBA-F2 (n = 297), and C57-F2 (n = 333) populations, we first plotted the frequency distributions of the 32 kHz ABR thresholds for each population. The 32 kHz ABR threshold distributions (Figure 1B-D) vary considerably, suggesting that the genetic factors that contribute to the differences in hearing within a population also vary between populations. As shown previously ([10]; reproduced in Figure 1B), the distribution of the C3H-N2 population is highly skewed and is best fit using a model for the sum of two Gaussian distributions (R^2^ = 0.97, n = 306). The skewing of the C3H-N2 distribution suggests that there is at least one allele influencing 32 kHz thresholds that has a relatively large effect in that population. In contrast, the 32 kHz frequency distribution of the CBA-F2 population is Gaussian (R^2^ = 0.96, n = 294) with a mean of 40 ± 5.97 (Figure 1C), suggesting that many genes of small and approximately equal effect influence 32 kHz thresholds in the CBA-F2 population. Finally, the distribution of the C57-F2 population (Figure 1D) appears to have a bimodal distribution with means at 37.9 ± 6.71 (R^2^ = 0.76, n = 238) and 70.7 ± 7.72 (R^2^ = 0.85, n = 82). The bimodal frequency distribution of the C57-F2 population suggests that there is a single locus responsible for a substantial difference in mean 32 kHz thresholds in that population.

**Figure 2:**
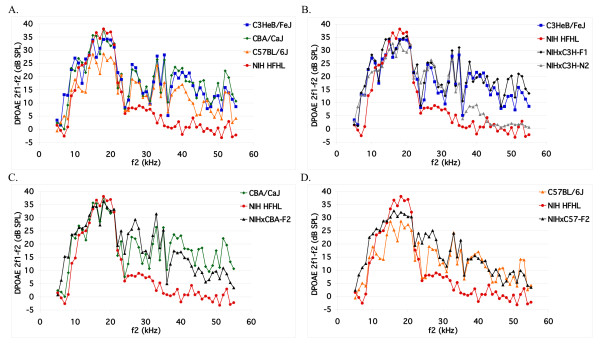
**Distortion product otoacoustic emissions.** Average DP-grams for mice (8–10 weeks old) in response to 5–55 kHz f2 inputs of 65 dB intensity. Distortion product amplitudes have been corrected to account for differences in noise floors between samples. (**A**) DP-grams of mice from each of the 4 parental strains: C3HeB/FeJ (n = 17), CBA/CaJ (n = 12), C57BL/6J (n = 12), NIH Swiss HFHL (n = 12). (**B**) DP-grams for the parental, F1 (n = 8), and N2 (n = 306) populations for the NIH Swiss HFHL x C3HeB/FeJ cross. (**C-D**) DP-grams for the parental and F2 populations for the (**C**) NIH Swiss HFHL x CBA/CaJ (n = 294) and (**D**) NIH Swiss HFHL x C57BL/6J crosses (n = 320). DPgrams of parental strains were redrawn in (**B-D**) for comparison.

To evaluate outer hair cell function and obtain quantitative phenotypic data across the entire frequency range of the cochlea, we measured distortion product otoacoustic emissions (DPOAEs). Average noise-floor-corrected emissions spectra for the four parental strains (C3HeB/FeJ, CBA/CaJ, C57BL/6J, and HFHL) are shown in Figure 2A. Eight-week-old C3HeB/FeJ and CBA/CaJ mice have robust 2f1-f2 emissions at all frequencies tested. Although C57BL/6J mice do appear to have slightly lower 2f1-f2 emissions at all frequencies than C3HeB/FeJ and CBA/CaJ mice (probably the result of a single C57BL/6J mouse that had no discernable emissions at any frequency), they still appear to have relatively normal 2f1-f2 emissions at 8 weeks of age. In agreement with our previous results, the HFHL mice have 2f1-f2 emissions approximately equal to those of the C3HeB/FeJ mice until the f2 input frequency reaches about 25 kHz, but have poor emissions beyond 25 kHz and basically no emissions when the f2 input is above 30 kHz.

The DPOAE graphs and high frequency emission average (HFEA) values for the three crosses further demonstrate the heritability of the HFHL phenotype (Figure 2B-D, Table 1). As expected for a heritable trait, the HFEA values for the C3H-N2 and the CBA-F2 populations are intermediate to those of their respective parental lines (Table 1) and the DPOAE spectra exhibit a similar relationship (Figure 2B, C). Additionally, the NIHxC3H-F1 population has an average DPOAE spectrum that is approximately equal to the C3HeB/FeJ spectrum (Figure 2B), indicating that the loss of high frequency emissions is due to recessive alleles. Interestingly, the C57-F2 population does not follow this pattern and has high-frequency hearing approximately equal to or slightly better than that of the C57BL/6J parental line (Figure 2D, Table 1). The C57-F2 population also appears to have better low-frequency hearing than either parental line, perhaps suggesting gene interactions.

The emissions intensities within the C3H-N2 and CBA-F2 populations appear to be highly variable at frequencies above 30 kHz (Data not shown). A few individuals from each cross exhibit substantially reduced emissions at frequencies as low as 20 kHz. At frequencies slightly higher than 30 kHz, the individual spectra become more variable, with many spectra lying obviously above the mean and others clearly below. The variation in the distributions becomes less pronounced at frequencies above about 45 kHz, probably due to an increase in noise during DPOAE measurement. Based on the variation in the DPOAE spectra, for each mouse we averaged the noise-floor-corrected 30–44 kHz DPOAE values in response to 65 dB SPL f2 input to create the variable, HFEA, that represented the quality of a mouse’s high-frequency emissions and was amenable to QTL analysis.

### Correlations in DPOAE data

To determine if correlations between emissions intensities produced by different frequencies differed depending on the strain, we evaluated the correlations between 2f1-f2 distortion product intensities independently for each strain. Significant correlations between 2f1-f2 distortion product intensities are present in all four parental populations (Additional file 1: Table S1, Additional file 2: Table S2, Additional file 3: Table S3, Additional file 4: Table S4). For the C3HeB/FeJ and CBA/CaJ strains, most of the significant correlations occur between 23–48 kHz frequencies (Additional file 2: Table S2, Additional file 3: Table S3). In both of these strains, the number of significant correlations exceeds the number expected by chance (C3HeB/FeJ: χ^2^ = 15.4, *p* < 0.0001; CBA/CaJ: χ^2^ = 44.1, *p* < 0.0001). In contrast, there were far fewer significant correlations between 2f1-f2 distortion product intensities in the HFHL mice. Furthermore, although initial examination of the C57BL/6J data suggested that a high percentage of the 2f1-f2 distortion product intensities were strongly correlated, these significant correlations were almost entirely the result of a C57BL/6J outlier that had no detectable 2f1-f2 distortion products for any f2 frequency. Since it is well documented that C57BL/6J mice have relatively normal DPOAE, especially at low frequency, until at least 3 months of age (see e.g. [12]), we eliminated the data from this outlier and reran the analysis for the C57BL/6J strain (Additional file 4: Table S4). Both the C57BL/6J strain and the HFHL line had fewer significant correlations (108 and 73, respectively) than the 127 significant correlations that are expected by chance due to the large number of tests (*p* > 0.05).

### Principal components analysis of DPOAE

**Figure 3:**
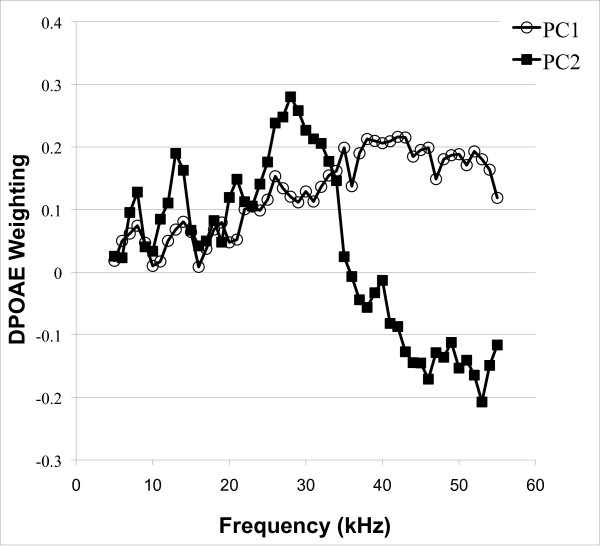
**Loading of principal components 1 and 2.** Loading of the first two principal components for DPOAE spectra are plotted as a function of the f2 stimulus frequency. The first component (PC1) has all positive loadings, indicating that it is a measure of the overall magnitude of emissions. The second component (PC2) has positive loading for low frequencies and negative loading for high frequency, indicating that it is a measure of the contrast between the magnitude of high frequency emissions and low frequency emissions.

To reduce the noise and complexity of the DPOAE spectra and produce new variables that best explain the variance in the population, we performed Principal Component Analysis on the C3H-N2 DPOAE data (see Figure 3). The first 5 principal components explain about 53% of the variation in DPOAE data in the population. The first principal component (PC1) explains about 25% of the variation in DPOAE and has positive loadings for each of the 51 variables (the 51 2f1-f2 distortion product intensities that were recorded for 65 dB SPL f2 input intensities across a range of frequencies), so PC1 may be considered a measure of the overall magnitude of DPOAEs (Figure 3). PC2 explains approximately 11% of the variation in DPOAE and has positive loadings for most of the variables representing frequencies below 34 kHz and negative loadings for the variables representing frequencies above 35 kHz (Figure 3). Thus, PC2 seems to be a measure of reduced high-frequency emissions relative to low-frequency emissions and may serve as a good indicator of HFHL. PC3 explains approximately 9% of the variation in DPOAE and has negative loadings for the variables representing 22–35 kHz frequencies and positive loadings for most other variables, so it seems to be a measure of reduced emissions at intermediate frequencies. PC4 and PC5 each account for only about 4% of the variation in DPOAE and appear to represent more complex contrasts. PC4 has positive loadings for the variables representing 33–42 kHz frequencies, but negative loadings for most other variables, indicating it is a measure of good intermediate frequency emissions. The loadings for PC5 seem to suggest that it is primarily an indicator of increased low frequency emissions in individuals with poor intermediate frequency emissions.

### Genetic analysis

Although 32 kHz ABR thresholds from the C3H-N2 population were used previously for QTL analysis [9], we reasoned that since the HFHL defect appears to be related to OHC function the DPOAE data might be more valuable than the ABR thresholds for identifying loci responsible for hearing impairment in our populations. We therefore used PC1 in a QTL analysis to identify genes influencing differences in overall magnitude of DPOAE in our C3H-N2 population (Table 2). The preliminary genome-wide QTL scan using the PC1 values identified two significant QTLs. A highly significant QTL (LOD = 4.6) that accounts for about 7% of the variation in PC1 lies near *rs6228386* on chromosome 7. The second significant QTL (LOD = 3.1) lies near *rs3709825* on chromosome 9 and accounts for about 5% of the variation in PC1. Furthermore, suggestive QTLs accounting for 2–3% of the variation were detected on chromosomes 6, 15, and 18.

**Table 2:** QTL influencing DPOAE in C3H-N2 mice

**Trait**	**Marker**	**Chr**	**LRS (LOD)**	***p*Value**	**Effect**
PC1	*rs6181382*	6	8.5 (1.84)	0.00353	3%
	*rs6228386*	7	21.2 (4.61)	<0.00001	7%
	*rs3709825*	9	14.1 (3.06)	0.00017	5%
	*rs3023429*	15	7 (1.51)	0.00813	2%
	*rs6358426*	18	8.7 (1.89)	0.00316	3%
HFEA	*rs13477127*	3	7.7 (1.67)	0.00547	3%
	*rs6228386*	7	24.2 (5.25)	<0.00001	8%
	*rs13480323*	9	19.4 (4.21)	0.00001	6%
	*rs3023429*	15	7.9 (1.97)	0.00482	3%
	*rs6358426*	18	9.1 (1.97)	0.00255	3%
5–9 kHz	*rs3658401*	5	12.7 (2.75)	0.00037	4%
	*rs13480208*	9	11.9 (2.58)	0.00057	4%
	*rs3698545*	14	11.5 (2.49)	0.00070	4%
10–14 kHz	*rs13477504*	3	7 (1.51)	0.00819	2%
	*rs13479082*	6	8.5 (1.84)	0.00347	3%
20–24 kHz	*gnf01.037.906*	1	7.2 (1.56)	0.00723	2%
	*rs3724711*	7	6.8 (1.47)	0.00901	2%
	*rs6207781*	9	8.6 (1.87)	0.00342	3%
25–29 kHz	*rs13476259*	1	7.5 (1.63)	0.00634	3%
	*rs6181382*	6	7.5 (1.63)	0.00626	3%
	*rs3724711*	7	16.4 (3.56)	0.00005	5%
	*rs6207781*	9	9.6 (2.08)	0.00199	3%
30–34 kHz	*rs13478952*	6	7.3 (1.58)	0.00674	2%
	*rs3724711*	7	23.8 (5.16)	<0.00001	8%
	*rs13480323*	9	7.2 (1.56)	0.00746	2%
35–39 kHz	*rs3688780*	3	11.4 (2.47)	0.00074	4%
	*rs6160140*	7	16.9 (3.67)	0.00004	6%
	*rs13480323*	9	18.4 (3.99)	0.00002	6%
	*rs3023429*	15	8.1 (1.76)	0.00432	3%
	*rs6358426*	18	7.3 (1.58)	0.00683	2%
40–44 kHz	*rs3688780*	3	11.1 (2.41)	0.00087	4%
	*rs13477669*	4	7.5 (1.63)	0.00603	3%
	*rs6228386*	7	13.8 (2.99)	0.00020	5%
	*rs13480323*	9	17.1 (3.71)	0.00004	6%
	*rs3023429*	15	8 (1.74)	0.00473	3%
45–49 kHz	*rs13482141*	14	9.4 (2.04)	0.00216	3%
	*rs3023429*	15	8.7 (1.89)	0.00317	3%
50–55 kHz	*rs3688780*	3	10 (2.17)	0.00155	3%
	*rs13459177*	15	7.6 (1.65)	0.00599	3%

Chr, chromosome; HFEA, High Frequency Emissions Average.

Although the results of the QTL analysis using PC1 were interesting, PC1 appears to be related to the magnitude of the 65 dB DPOAE spectra across all frequencies, while the most intriguing feature of the hearing loss in our populations is its frequency specificity. Therefore, we elected to search for QTLs using PC2, PC3, and HFEA, which subdivided the frequency spectra. No significant QTLs were detected for PC2 and PC3, but the analysis of HFEA revealed 2 highly significant QTLs (Table 2). A highly significant QTL (LOD = 5.2) that accounted for about 8% of the variation in HFEA values was located near *rs6228386* on chromosome 7. Another QTL (LOD = 4.2) accounted for about 6% of the variation in HFEA and was located on chromosome 9 near *rs13480323*. Additional loci suggestive of QTLs were identified on chromosomes 3, 15, and 18. These putative QTLs were each responsible for about 3% of the variation in HFEA values.

**Figure 4:**
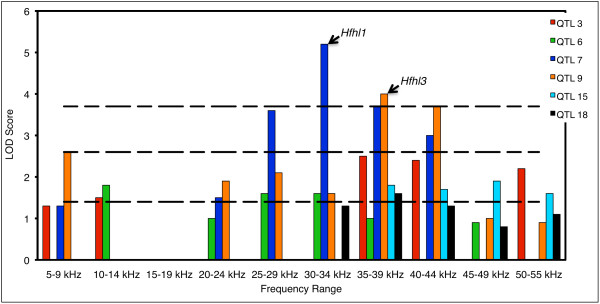
**Frequency specificity of C3H-N2 QTLs.** The heights of the vertical bars indicate the LOD scores calculated for QTLs that affect one or more of the 10 segments into which the DPOAE spectrum was divided. Only those QTL that were first identified in the marker regression analyses of the C3H-N2 population using PC1 or HFEA as the quantitative trait are shown. Each color represents a particular locus and the number given for the QTL indicates the chromosome on which the QTL was detected. Dashed lines indicate the permutation derived genome-wide significance levels for suggestive, significant, and highly significant linkage. No bars are shown in cases for which the *p* value of the LOD score exceeded 0.05.

To determine whether different genes affect the emissions values depending on the frequency evaluated, we decomposed the 65 dB 2f1-f2 distortion product intensity data and created a series of average DPOAE intensities calculated from frequency ranges of approximately 5 kHz each (5–9 kHz, 10–14 kHz, 15–19 kHz, 20–24 kHz, 25–29 kHz, 30–34 kHz, 35–39 kHz, 40–44 kHz, 45–49 kHz, and 50–55 kHz) of the 65 dB DPOAE frequency spectra. Then, we ran marker regressions on the average DPOAE values calculated for the ten 5 kHz segments. A number of suggestive and significant QTLs were detected (Table 2). All of these QTLs affected only a subset of the 5 kHz segments. However, the QTLs generally appeared to affect multiple segments that tended to be clustered together (Figure 4). A QTL on chromosome 7 near *rs6160140* significantly affected the segments encompassing the entire 25–44 kHz frequency range and a QTL on chromosome 9 near *rs13480323* significantly affected segments in the 35–44 kHz frequency range. Loci on chromosome 3 and 15 were suggestive of QTLs with effects on the 35–55 kHz frequency range.

**Figure 5:**
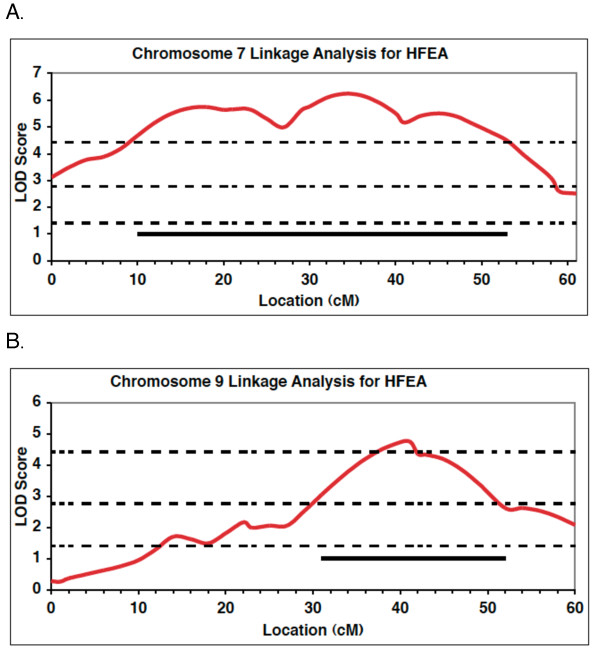
**Interval maps of HFEA QTLs on chromosomes 7 and 9.** Interval mapping plots for QTL affecting the HFEA trait on (**A**) chromosome 7 and (**B**) chromosome 9 in the C3H-N2 population. Dashed lines indicate the permutation derived genome-wide significance levels for suggestive, significant, and highly significant linkage. Bars indicate the 1.5 LOD support interval.

To more accurately identify the locations of the two most significant QTLs affecting DPOAEs, we performed composite interval mapping for HFEA on both chromosome 7 and chromosome 9 (Figure 5). In these analyses, we included the highly significant locus located on the opposite chromosome as background. The QTL on chromosome 7 encompasses much of the chromosome and spans the *Hfhl1* interval that we detected previously using ABR thresholds [10]. The QTL on chromosome 9 appears to lie at approximately 40 ± 10 cM.

Although we were unable to perform genome-wide QTL mapping for the CBA-F2 and C57-F2 populations, we were able to genotype them at a few loci for association mapping. To confirm our earlier analysis, the mice from the CBA-F2 and C57-F2 populations were genotyped at loci on chromosomes 7 and 9 that are located near the QTLs that we detected in the C3H-N2 population (see Table 3). Furthermore, we genotyped each population at loci on chromosome 8 that are located near *Hfhl2*, a locus that was found to affect only 32 kHz ABR thresholds in a previous study using NIH Swiss mice (Table 3). Finally, we genotyped both populations at loci all along chromosome 10 in order to account for effects of the *Cdh23*^*ahl*^ mutation in the C57-F2 cross (Table 3).

**Table 3:** Loci Genotyped in C57-F2 and CBA-F2 Crosses

**Cross**	**Chr**	**Locus**	**Location (Mbp)**	**Corresponding** **C3H-N2 SNP Interval**
C57-F2	7	*D7MIT83*	59.0	rs13479325*-rs6279696
	8	*rs13479840*	78.3	rs3667255-rs6296891*
		*rs13479929*	97.3	rs3661882*
	9	*rs13480341*	90.8	rs13480325*-rs3088463
	10	*rs13480480*	7.5	CEL10_3989939*-CEL10_20959105
		*rs3089912*	20.7	CEL10_20959105*-rs6391751
		*rs13480550*	25.0	CEL10_20959105*-rs6391751*
		*rs13480621*	59.9	*rs6391751-rs3682523*
		*rs29365220*	81.7	rs3682523-rs8258353*
		*rs13480720*	96.8	rs13480720*
		*rs13480777*	115.6	*rs13480720*
CBA-F2	7	*rs8255275*	46.0	*rs6228386-rs6160140*
		*rs3680765*	56.6	rs6160140*-rs13479325*
		*rs3719301*	64.2	*rs13479325-rs6279696*
	8	*rs13479840*	78.3	rs3667255-rs6296891*
		*rs13479916*	94.3	rs6296891-rs3661882*
	9	*rs13480313*	84.0	rs13480208-rs13480323*
	10	*rs3089912*	20.7	CEL10_3989939-CEL10_20959105*
		*rs13480550*	25.0	CEL10_20959105*-rs6391751*
		*rs13480581*	38.7	*rs6391751-rs3682523*
		*rs13480652*	74.1	rs3682523*-rs8258353
		*rs13480720*	96.8	rs13480720*
		*rs13480777*	115.6	*rs13480720*

* SNP is within 5 Mbp of the SNP evaluated in the C57-F2 or CBA-F2 population. Chr, chromosome; Mbp, mega base pairs.

We detected significant QTLs on both chromosome 7 and chromosome 9 in the CBA-F2 population, confirming the results of the C3H-N2 population (Table 4). We also detected a chromosome 7 QTL in the C57-F2 population. However, its effects were small, probably because of the highly significant QTL of large effect that is located on chromosome 10 (Table 4) that probably represents the effect of the *Cdh23*^*ahl*^ allele that is known to be present in the C57BL/6J strain [13].

**Table 4:** QTL influencing hearing in CBA-F2 and C57-F2 mice

**Line**	**Trait**	**Chr**	**Marker**	**LRS (LOD)**	***p*Value**	**Effect**
C57-F2						
	32 kHz ABR	10	*rs13480621*	280.2 (60.7)	<0.00001	57%
	HFEA	10	*rs13480621*	311.6 (67.6)	<0.00001	63%
		7	*D7MIT83*	10.2 (2.2)	0.00614	2%
	PC1	10	*rs13480621*	259.9 (56.4)	<0.00001	56%
	PC2	10	*rs13480621*	25.8 (5.6)	<0.00001	8%
	PC3	10	*rs39365220*	12.0 (2.6)	0.00250	4%
	5–9 kHz	10	*rs3089912*	19.2 (4.2)	0.00007	6%
	10–14 kHz	10	*rs13480621*	20.4 (4.4)	0.00004	6%
	15–19 kHz	10	*rs13480621*	35.3 (7.7)	<0.00001	11%
	20–24 kHz	10	*rs13480621*	143.0 (31.0)	<0.00001	36%
	25–29 kHz	10	*rs13480621*	202.6 (43.9)	<0.00001	47%
	30–34 kHz	10	*rs13480621*	250.1 (54.3)	<0.00001	55%
	35–39 kHz	10	*rs13480621*	290.1 (62.9)	<0.00001	60%
		7	*D7MIT83*	11.4 (2.5)	0.00336	2%
	40–44 kHz	10	*rs13480621*	176.2 (38.2)	<0.00001	43%
	45–49 kHz	10	*rs13480621*	178.1 (38.6)	<0.00001	22%
	50–55 kHz	10	*rs13480621*	87.8 (19.0)	<0.00001	24%
CBA-F2						
	HFEA	7	*rs3680765*	19.7 (4.27)	0.00005	7%
		9	*rs13480313*	10.9 (2.4)	0.00425	4%
	PC1	7	*rs3680765*	18.5 (4.0)	0.00010	6%
	PC3	7	*rs3719301*	10.9 (2.4)	0.00428	4%
	35–39 kHz	7	*rs3680765*	22.4 (4.9)	0.00001	7%
	40–44 kHz	7	*rs3680765*	13.4 (2.9)	0.00122	5%
		9	*rs13480313*	11.0 (2.4)	0.00415	4%
	45–49 kHz	7	*rs3680765*	15.1 (3.3)	0.00053	5%
	50–55 kHz	7	*rs8255275*	11.4 (2.5)	0.00346	4%

Chr, chromosome; HFEA, High Frequency Emissions Average.

In addition to testing for single locus effects, we also tested whether any two loci interacted to affect 32 kHz ABR thresholds, HFEA, or PC1-3 in each of the populations. This was done using very stringent conditions in order to reduce the possibility that the large number of tests might give spurious positive results. Under these stringent criteria, no significant interactions were detected in any of our populations.

## Discussion

In this study, we produced several crosses to investigate the genetic basis of impaired high frequency hearing in the HFHL line of mice. To fully explore the entire range of hearing, we used a number of methods for quantifying and dissecting the DPOAE data from our populations. These methods produced different values that could be used in QTL mapping. We detected a QTL on chromosome 7 (*Hfhl1*) and a QTL on chromosome 9 (*Hfhl3*) that significantly affect hearing. Furthermore, our analysis allowed us to refine the frequency ranges that these two QTLs affect and to detect additional putative QTLs with suggestive effects on DPOAE that were undetectable using ABR thresholds. The QTLs were scattered throughout the genome, varied considerable in effect size, and appeared to have frequency-specific effects in many cases.

### QTL effects

Several SNPs on chromosome 7 were found to have significant effects on hearing in the whole genome scan of the C3H-N2 population. In general, these SNPs lie relatively near one another on the chromosome and are contained within the confidence interval of the rather broad QTL peaks observed for each trait. These loci probably all represent the same QTL on chromosome 7, although it is possible that linked loci are involved. This presumptive chromosome 7 QTL affected multiple measures of hearing in both the C3H-N2 and CBA-F2 population and accounted for as much as 8% and 7% of the observed variation in DPOAE, respectively. Although the effects were much less noticeable in the C57-F2 population, it was detected in that population as well, despite the potentially confounding effects of the highly significant chromosome 10 QTL. The chromosome 7 QTL that we detected in this study is in the same region as the *Hfhl1* locus that we previously identified as being responsible for increased 32 kHz ABR thresholds in both the HFHL line and in another line of mice produced from the NIH Swiss stock [10]. Our results confirm the effect of the *Hfhl1* locus and confine its effect to the 25–44 kHz region of the tonotopic map.

Several linked loci on chromosome 9 were found to significantly affect a number of DPOAE values in the C3H-N2 population. These chromosome 9 loci, like those on chromosome 7, probably all represent a single QTL (*Hfhl3*). Although undetectable using ABR thresholds, *Hfhl3* explains as much as 6% of the observed variation in the DPOAE of the C3H-N2 population. Interestingly, our analysis indicates that the effect of *Hfhl3* is restricted to the 35–44 kHz region of the tonotopic map. Although *Hfhl3* was found to have moderate effects on multiple measures of hearing in both the C3H-N2 and the CBA-F2 lines, the single locus genotyped in the same region of chromosome 9 in the C57-F2 cross did not significantly affect hearing. This could be due to reduced power to detect a QTL of moderate effect in the presence of the chromosome 10 locus of large effect, or due to insufficient linkage between the chromosome 9 SNP that was genotyped and the actual locus affecting hearing, although the locus chosen is in the middle of the confidence interval for the QTL in the C3H-N2 population. Interestingly, in our previous study using HFHL mice, a suggestive QTL influencing 32 kHz ABR thresholds was detected at *rs13480208* (at 55 Mbp on chromosome 9, Build 34) in the HFHL-N2 population [10]. This is close to the location of the *rs13480323* (at 84 Mbp) and *rs13480313* (at 88Mbp) SNPs that significantly affected DPOAE in this study.

The *Hfhl1* and *Hfhl3* loci only explain a portion of the variation in high-frequency hearing observed in the C3H-N2 and CBA-F2 populations. Additional loci of small effect and epistasis probably account for much of the remainder of the variation and may also explain the severity of the hearing phenotype observed in our HFHL line compared to the relatively mild phenotypes exhibited in the C3H-N2 and CBA-F2 populations. By dividing the frequency spectra of the C3H-N2 population into smaller segments, we detected a number of additional loci that potentially account for residual variation in the C3H-N2 population, supporting the role of additional loci in the HFHL phenotype. However, epistatic effects probably play a role as well since abundant epistasis has been documented in studies of a number of complex traits [14-16]. Additional genotyping and an in-depth analysis of epistasis in the CBA-F2 population could greatly increase our understanding of the genetic architecture of the HFHL phenotype.

The QTL with the largest effect in the C57-F2 population was on chromosome 10 near *rs13480621*. This QTL accounted for over 50% of the variation in 32 kHz ABR thresholds, HFEA, and PC1 in the C57-F2 population, but was not detected in the other two crosses, suggesting that the allele responsible segregated in the C57BL/6J P0 strain. Given its location and the population in which it was detected, the QTL on chromosome 10 near *rs13480621* is almost certainly the result of the *Cdh23* locus that is known to reside in the same region of chromosome 10. The *Cdh23*^*ahl*^ allele contributes to age-related hearing loss in many inbred strains, including the C57BL/6J strain [13] from which the C57-F2 population was produced.

The failure to detect a chromosome 7 QTL for ABR thresholds in the C57-F2 population may simply be because the large effect of the chromosome 10 QTL obscured its effects. Decreased power could also explain the failure to detect QTL affecting 32 kHz ABR thresholds in the CBA-F2 population. However, it is possible that the CBA-F2 population possesses one or more genetic modifiers that attenuate the effects of the reduced emissions such that ABR thresholds are normal despite the reduced emissions. Genetic background and genetic modifiers have been found to attenuate the effects of a number of other genes that affect hearing in mice [17]. We did not detect any genetic interactions in this study, but the power to detect them is low and we used stringent criteria to limit the chance of detecting false positives.

### Analysis of DPOAE spectra

In this study, the chromosome 7 QTL had a significant effect on PC1, an indicator of the overall magnitude of DPOAE, suggesting that the effects of *Hfhl1* might not be specific to high-frequency hearing. However, by dividing the DPOAE into 5-kHz segment, we showed that the chromosome 7 QTL has no effect on frequencies below 20 kHz, but significantly affects DPOAEs between 25 kHz and 44 kHz. In fact, the effects of this QTL are greatest for 30–39 kHz frequencies in all three of our populations. Thus, it appears that this effect on PC1 is primarily, if not completely, due to its effect on the high-frequency portion of the spectrum. These results support the frequency-specific role of *Hfhl1* that the ABR data suggested [10].

Our analysis using HFEA produced very similar results to the analysis using PC1, supporting the validity of using our somewhat arbitrary divisions of the DPOAE spectra as quantitative traits for mapping. Dividing the DPOAE spectra into segments for analysis of frequency-specific QTLs is further validated by the results of the correlation matrices of the DPOAE data which seem to support our hypotheses that emissions intensities produced in response to similar frequencies would be more correlated than those produced in response to less similar frequencies and that any alleles that decrease hearing would reduce those correlations. By dividing the emissions spectra, we found that different QTLs affected emissions for different frequencies and that each only affected a portion of the entire frequency spectrum (see Table 2). These results should not be over-interpreted to mean that the QTLs have discrete boundaries of expression in the cochlea or that they are active in specific tonotopic regions only. The effects of these QTLs might be detectable at particular frequencies because their functions are less critical in seemingly unaffected regions. However, the QTL boundaries could also be the result of cochlear mechanics that accentuate hearing deficits caused by those QTLs in certain regions, or because our power to detect QTL effects is lower in other cochlear regions.

It is somewhat surprising that our analysis did not produce additional principal components for which we could detect QTLs. This is particularly true for PC2 because the loadings indicated that it was a measure of the contrast between an individual’s high-frequency and low-frequency emissions, which would appear to best describe the difference between HFHL and normal hearing mice. However, perhaps there was simply insufficient power to detect QTLs accounting for the variation remaining in the population after the effects of PC1 were removed. This would be especially true if PC2 was the result of gene interactions.

### Implications of analysis

The QTLs discovered in this study affect hearing via attenuation of outer hair cell function in the base of the cochlea. Dysmorphic hair bundles are common in hearing loss [18] and the high frequency (basal) portion of the cochlea seems to be most sensitive to damage. However, our previous morphological analyses did not reveal abnormalities in the OHC hair bundles that coincided with the onset of hearing loss [10]. Furthermore, mutations that affect hair bundle morphology often produce severe or progressively worsening hearing loss [10,18], while hearing loss is mild and relatively stable in the NIH Swiss mice [10]. Similarly, mutations that cause changes in endolymph composition can alter the endocochlear potential and disrupt OHC function by preventing mechano-electrical transduction [19]. Although ion concentrations were not tested, the endocochlear potential is normal in NIH Swiss mice [10].

The early-onset, frequency-specific, and stable nature of the hearing loss in NIH Swiss mice suggests that the loci responsible for the phenotype may have a developmental role that differs in importance along the cochlear duct. A number of gene products have been shown to have a graded expression along the organ of Corti [4], so it is conceivable that a hypomorphic allele of one of these genes might have a mild and frequency-specific effect on hearing. On the other hand, theoretical modeling demonstrated that cochlear shape might affect cochlear response, at least for low frequency sounds [20]. Similarly, it is possible that sensitivity to high-frequency sounds could also be reduced by morphological or compositional changes to the cochlear duct, tectorial membrane, or basilar membrane since properties of these structures vary tonotopically [1] and data suggests that cochlear function is dependent on the mechanical properties of these cochlear structures [21]. Although gross morphology looked normal in NIH Swiss mice [10], a more detailed analysis of the relative size and shape as well as the molecular composition of these mechanically important components is needed.

Although most mapping studies have used ABR thresholds to identify QTLs responsible for differences in hearing, others have used CAP thresholds [22,23], and at least one previous study used DPOAE amplitude [24]. Using either PC1 or HFEA to summarize the DPOAE values we were able to detect a chromosome 7 QTL in all of our populations even though we did not detect the QTL in either the NIHxC57-F2 population or the NIHxCBA-F2 population when ABR thresholds were used. Similarly, *Hfhl3* was only detected when DPOAE values were used in the analysis. Furthermore, using DPOAE data we were able to detect a number of putative loci of small effect that were not recognized in our previous analysis using ABR thresholds [10]. Most interestingly, by using DPOAE data, refine the regions affected by *Hfhl1* and *Hfhl3* to limited portions of the entire hearing spectrum. These results suggest that DPOAE measurements coupled with QTL analyses should be given serious consideration when designing experiments to investigate the molecular basis of hearing loss, particularly since DPOAEs are sometimes able to detect changes in hearing that are undetectable via ABR [25] and DPOAE efficiently provides a more thorough map of the cochlea than standard ABR protocols.

## Conclusions

The non-progressive nature of the HFHL phenotype makes the HFHL line of mice useful for studying the genetic basis of frequency-specific hearing loss. By crossing the HFHL line with different strains, we identified two QTLs that affect emissions intensity over a limited portion of the DPOAE spectrum and other putative loci with similarly frequency-specific roles. These results support the hypothesis that frequency-specific hearing loss is the result of regionally specific gene activity along the organ of Corti, and suggest that DPOAE measurements combined with QTL mapping is a useful strategy for determining the molecular factors underlying basal to apical differences in hearing acuity.

## Methods

### Population

NIH Swiss mice were obtained from Charles River Laboratories (Wilmington, MA). C3HeB/FeJ, CBA/CaJ and C57BL/6J mice were obtained from The Jackson Laboratory (Bar Harbor, Maine). High frequency hearing loss (HFHL) mice were generated by selectively breeding NIH Swiss mice that had good hearing at low frequency, but poor high-frequency hearing. This was followed by several (12–15) generations of inbreeding [10]. Although the line was not entirely isogenic at this stage, the HFHL phenotype was 100% penetrant with consistent expression in the HFHL line, so we considered the mice sufficiently inbred at this point to begin our experiment. A few F12-F15 HFHL mice were crossed to C3HeB/FeJ, CBA/CaJ and C57BL/6J mice to create three different F1 populations. Several (HFHL x C3HeB/FeJ) F1 mice were backcrossed to HFHL mice to produce a population of [(HFHL x C3HeB/FeJ) x HFHL] N2 mice, hereafter C3H-N2 mice (n = 306). Additionally, randomly selected (HFHL x CBA/CaJ) F1 and (HFHL x C57BL/6J) F1 mice were each intercrossed to produce two F2 populations, hereafter designated CBA-F2 (n = 294) and C57-F2 (n = 320), respectively. The care and use of animals were performed in compliance with the guidelines at the National Institutes of Health and approved by the Animal Care and Use Committee of the NINDS/NIDCD.

### Auditory brain stem response measurements

Auditory-evoked brain stem response (ABR) measurements were made with the Smart-EP version 10 (Intelligent Hearing System, IHS; Miami, Florida) computer-aided evoked potential system. The system was modified with high frequency transducers to permit high frequency measurements. Electrodes were inserted subdermally at the vertex of the cranium (active) and ventrolaterally to the right (reference) and the left (ground) ears. The instrument generated specific acoustic stimuli and then measured and displayed the evoked brainstem responses of anesthetized mice. A series of acoustic stimuli were delivered from the high-frequency transducers to the right outer ear canal. Mice were presented with a 32 kHz pure tone that was varied in intensity from 100 dB SPL to 10 dB SPL in 5 dB increments. Each intensity level was presented at a rate of 19.1 times/s for 350 sweeps. The lowest intensity producing two consistent characteristic waveforms was recorded as the 32 kHz ABR threshold.

### Distortion product otoacoustic emission measurements

DPOAEs were measured with National Instruments (NI) LabView 8.6 software installed on a PC. An NI PCI-4461 Dynamic Signal Analyzer (DSA) sound card and Clarion SRU310H high frequency dome tweeters were used to deliver two pure tones into the outer ear canal of each mouse. The two tones, f1 and f2, had a fixed f2/f1 ratio of 1.25 and presentation intensities of f2 = f1- 10 dB. The f2 tones were varied from 5 kHz to 55 kHz over a range of intensities (15 dB SPL to 85 dB SPL in 10 dB increments). The amplitudes of the 2f1- f2 distortion products were recorded along with the corresponding noise floor measurements. All calculations performed on DPOAE were performed on noise floor corrected amplitudes.

### Analysis of DPOAE spectra

In order to extract pertinent information from the large amount of data provided by the DPOAE measurements, we analyzed the data in several ways. First, we reasoned that good frequency discrimination might require proximal portions of the organ of Corti to be more developmentally and functionally integrated than regions that are more distal. Thus, we hypothesized that within a particular strain, the intensity of the 2f1-f2 distortion products would be more correlated for similar f1 frequencies than for frequencies that were farther apart. To test this, we calculated the correlation coefficients between the 2f1-f2 distortion products resulting from different 65 dB f2 tones and looked for patterns among the correlation coefficients. Furthermore, we suspected that alleles that affect hearing could alter the correlations between the distortion products intensities produced in response to stimuli of different frequencies, particularly if the genes involved were frequency-specific. We hypothesized that any alleles with frequency-specific effects would decrease the correlations between the distortion products of the affected cochlear regions and unaffected frequencies. To evaluate this hypothesis, we compared the correlations between all 2f1-f2 distortion products produced in response to 65 dB intensity f2 inputs in the 4 parental lines and in the first generation (F1) offspring of the three crosses. Finally, we performed Principal Component Analysis (PCA) to determine which aspects of the frequency spectra best differentiated between the individuals within each HFHL cross.

A substantial number of mice from the N2 and F2 populations had normal 32 kHz ABR thresholds but seemed to exhibit abnormally high frequency DPOAE spectra. Since hearing loss in HFHL mice appears to be related to OHC function [10] and the neuronal activity recorded by ABR is several physiological steps removed from OHC activity, we reasoned that the DPOAE results might provide a better quantitative measure for QTL analysis than the ABR thresholds. Therefore, we evaluated the DPOAE curves in order to devise a quantitative value that was amenable to QTL analysis but reflected the readily observable differences in DPOAE among mice in our populations. Variability in noise level and random, bidirectional spikes in the recorded 2f1-f2 distortion product intensities precluded the determination of a clear frequency threshold for an individual’s DPOAE. Similarly, the 2f1-f2 distortion product intensity at any particular frequency was untenable as a meaningful quantitative measure of emission quality for an individual. However, mice with normal emissions could be differentiated from mice with impaired emissions by comparing the 2f1-f2 distortion product intensities generated over an isolated range of high frequency (>30 kHz) f2 inputs of high intensity. Since mice with hearing thresholds greater than 60 dB SPL are generally considered to be hearing-impaired, we chose to use the results of the 65 dB f2 stimulus intensity. For each mouse, we averaged the noise-floor corrected 2f1-f2 intensities produced over the range of frequencies (30–44 kHz) with the most conspicuous difference between individuals. These High Frequency Emissions Averages (HFEA) served as a quantitative measure of the quality of an individual’s DPOAE.

To further dissect and summarize the DPOAE data and to produce less subjective quantitative values that were appropriate for linkage mapping, we calculated principal components for the 2f1-f2 distortion product intensities from the three crosses. Principal components analysis converts a correlation matrix into a smaller set of orthogonal values (the principal components) that best describe the patterns in the original data [26]. In this analysis, we used the first three principal components (PC1-3) as additional quantitative traits for QTL analysis in each population.

We also reasoned that since OHC physiology and activity mirror the frequency gradient of the cochlea, our DPOAE data might provide us with an opportunity to discover some of the loci involved in producing basal to apical differences in cochlear properties. We hypothesized that if frequency-specific differences in hearing result from different genes being active in different regions of the cochlea, then different loci (QTL) would be detected for different frequency ranges. Therefore, we divided the DPOAE frequency spectra into 5 kHz segments (5–9 kHz, 10–14 kHz, 15–19 kHz, 20–24 kHz, 25–29 kHz, 30–34 kHz, 35–39 kHz, 40–44 kHz, 45–49 kHz, and 50–55 kHz), obtained noise floor corrected average DPOAE values for each of these segments, and used each of these averages as a quantitative trait for mapping QTL.

### Genetic analysis

DNA was extracted from tail clips using DNeasy® Blood and Tissue kits (Qiagen, Valencia, CA). Samples from C3H-N2 mice were adjusted to approximately 100 ng/μl (40–130 ng/μl) for genome-wide genotyping at The Partners Center for Personalized Genetic Medicine (Cambridge, MA). These C3H-N2 mice and randomly selected P0 and F1 mice were genotyped at 337 SNPs (single nuceotide polymorphisms) spaced an average of 6.5 Mb apart. Of these 337 SNPs, 145 were useful in our analysis. Aliquots from the C3H-N2 samples and DNA samples from CBA-F2 and C57-F2 mice were diluted to approximately 10 ng/μl (1–20 ng/μl) for additional genotyping using TaqMan® SNP Genotyping Assays with a StepOnePlus Real-Time PCR Thermocycler (Applied Biosystems). For genomic regions where the available TaqMan® SNP Genotyping Assays were not suitable for genotyping a particular cross, mice were genotyped at microsatellites for which the two parental strains were found to be polymorphic. For microsatellite genotyping, the microsatellite regions of the DNA samples were amplified via PCR with AmpliTaq® DNA Polymerase (Applied Biosystems) mixed with standard concentrations of dNTP, 10x PCR reaction buffer, and forward and reverse primers in molecular biology grade water. For PCR amplification, samples were denatured (95 ° C for 1 min), then subjected to 50 cycles consisting of denaturation (94 ° C for 45 s), annealing (55 ° C for 1 min), and extension (72 ° C for 1 min), followed by a final extension step (72 ° C for 10 min). For genotype determination, 1 μl of each PCR reaction was mixed with 8.9 μl of formamide and 0.1 μl of GeneScan^TM^ 500ROX^TM^ size standard and electrophoresed on a 3730xl capillary DNA analyzer (Applied Biosystems). Data were analyzed using ABI^TM^ Prism GeneMapper^TM^ v 3.5 software. For F2 individuals for which the two homozygous genotypes were difficult to distinguish from the electropherograms, a second run was performed on each homozygote using a mix consisting of equal concentrations of the sample DNA and DNA from one of the parental strains. If this second run resulted in a homozygous genotype, the sample was declared to possess the same genotype as the parental strain used in the mix. If the second run resulted in a heterozygous genotype, the sample’s genotype was recorded as having the opposite parental strain’s genotype.

### QTL analysis

Previously, we performed a whole-genome QTL analysis using the ABR thresholds obtained from approximately 300 C3H-N2 mice when they were 8 weeks old [10]. To more fully explore the genetics underlying high-frequency hearing loss, the same mice were used in this study to identify QTL that significantly affect DPOAE. Genotypes at 145 SNPs and the corresponding DPOAE measures (PC1-3, HFEA, and averages obtained for each 5 kHz segment of the 65 dB frequency spectra) for each mouse were imported into Map Manager QTXb20. Map Manager QTXb20 was used to perform least-squares linear regressions and generate LRS (likelihood ratio statistic) scores [27]. 1000 permutations of the data were generated and evaluated by linear regression to establish significance thresholds for the LRS scores generated by the analysis [28]. LRS scores greater than 37, 95, or 99.9% of the permutation-derived values (equivalent to genome-wide *p* values of 0.63, 0.05, and 0.001) were considered suggestive, significant, or highly significant evidence of QTL, respectively [29]. LRS scores were converted to LOD scores (LRS/2ln(10) = LOD) by dividing by 4.61.

We next performed composite interval mapping on each chromosome for which a putative QTL was detected in the marker regression analysis. This allowed us to more accurately estimate the locations of the QTL and to determine support intervals [30]. We included the most significant unlinked QTL from the marker regressions as background loci in each of these analyses. Furthermore, we tested for associations between each trait and each pair of marker loci to identify potential gene interactions (i.e. epistatic effects). We only considered interactions significant if the effect of the combined loci were significant at the *p* = 10^-5^ level and the interaction effect was also significant at the permutation derived *p* = 0.01 level.

We performed a similar linkage analyses for our CBA-F2 and C57-F2 populations. Significance thresholds for these analyses were obtained using permutation tests and via the “Quick Test” option of QTXb20. The more stringent threshold value of the two calculated for each trait was selected as the actual threshold value. Since only a portion of the genome was mapped, we did not calculate significance levels for suggestive QTL. Threshold values exceeding the *p* = 0.05 threshold value were considered evidence of a QTL and those exceeding p = 0.001 were considered highly significant. Furthermore, 32 kHz ABR thresholds were included in the phenotypic data, but the genotypic data was limited to SNPs located in regions that had already been putatively identified as containing QTL or suggestive QTL in our original analysis or in the current analysis of the C3H-N2 population. SNPs all along chromosome 10 were also genotyped so that any effects of *Cdh23* mutations could be eliminated and potential interactions with chromosome 10 loci could be evaluated. ABR thresholds were included for comparison to the C3H-N2 results obtained previously. The loci genotyped in the C57-F2 and CBA-F2 populations are listed on Table 3, along with their locations and the closest corresponding loci genotyped in the C3H-N2 population.

### Statistical analyses

Differences between groups were assessed by one-way ANOVA followed by Bonferroni corrected post-tests after determining that they had equal variances and Gaussian distributions. For comparisons between groups that failed to meet the requirements of ANOVA, Kruskal-Wallis tests with post-tests were performed. GraphPad Prism 4.0b software was used to calculate means, plot the data, and compute *p* values for the comparisons.

Correlation coefficients and PCs were calculated using the multivariate option of JMP^TM^ Software version 5.0.1.2 from SAS Institute. Pearson Product–moment Correlation coefficients were tested for significance using the Pairwise Correlations option. Because we expected the 2f1-f2 distortion product amplitudes to be positively correlated, we used one-tailed tests to assess the significance of these correlations. However, since many correlations were evaluated in each population and a proportion of these correlations (10%) are expected to be significant simply due to chance, we used χ^2^ tests to determine whether or not the proportion of significant correlations was greater than expected due to chance.

### Description of additional data files

The following additional data are available with the online version of this paper. Additional data file 1–4 are tables of the correlation coefficients between the 2f1-f2 distortion products resulting from different 65 dB f2 tones in the HFHL line, the C3HeB/FeJ, the CBA/CaJ, and the C57BL/6J strains.

## Abbreviations

ABR, Auditory evoked brain stem response; DPOAE, Distortion product otoacoustic emission; GWAS, Genome wide association studies; HFEA, High frequency emission average; HFHL, High frequency hearing loss; LRS, Likelihood ratio statistic; OHC, Outer hair cell; PCA, Principal components analysis; QTL, Quantitative trait locus; SNHL, Sensorineural hearing loss.

## Competing interests

The authors declare that they have no competing interests.

## Authors’ contributions

JMK carried out phenotypic and genetic analysis, participated in design of the study, performed statistical analysis, and drafted the manuscript. KNT Conceived of the study, participated in its design and coordination, and helped to draft the manuscript. All authors read and approved the final manuscript.

## Supplementary Material

Additional file 1 **Table S1. **Correlations between 2f1-f2 emissions intensities produced in response to different f2 input frequencies in the HFHL line. Bold values are significant. Click here for file

Additional file 2 **Table S2. **Correlations between 2f1-f2 emissions intensities produced in response to different f2 input frequencies in the C3HeB/FeJ strain. Bold values are significant. Click here for file

Additional file 3 **Table S3. **Correlations between 2f1-f2 emissions intensities produced in response to different f2 input frequencies in the CBA/CaJ strain. Bold values are significant. Click here for file

Additional file 4 **Table S4. **Correlations between 2f1-f2 emissions intensities produced in response to different f2 input frequencies in the C57BL/6J strain. Bold values are significant. Click here for file
